# CD151-mediated adhesion is crucial to osteosarcoma pulmonary metastasis

**DOI:** 10.18632/oncotarget.11380

**Published:** 2016-08-19

**Authors:** Zhuoying Wang, Chongren Wang, Zifei Zhou, Mengxiong Sun, Chenghao Zhou, Jian Chen, Fei Yin, Hongsheng Wang, Binhui Lin, Dongqing Zuo, Suoyuan Li, Lijin Feng, Zhenfeng Duan, Zhengdong Cai, Yingqi Hua

**Affiliations:** ^1^ Department of Orthopedics, Shanghai General Hospital, Shanghai Jiao Tong University School of Medicine, Shanghai, 200080, China; ^2^ Shanghai Bone Tumor Institution, Shanghai, 201620, China; ^3^ Department of Pathology, Shanghai Tenth People's Hospital, Tongji University School of Medicine, Shanghai, 200072, China; ^4^ Sarcoma Biology Laboratory, Department of Orthopaedic Surgery, Massachusetts General Hospital and Harvard Medical School, Boston, MA, 02114, USA

**Keywords:** osteosarcoma, tetraspanin, CD151, metastasis, adhesion

## Abstract

CD151, a tetraspanin family protein involved in cell-cell and cell-extracellular matrix interaction, is differentially expressed in osteosarcoma cell membranes. Thus, this study aimed to investigate the role of CD151 in osteosarcoma metastasis. We analyzed CD151 expression in patient tissue samples using immunohistochemistry. CD151 expression was also silenced with shRNA in osteosarcoma cells of high metastatic potential, and cell adhesion, migration and invasion were evaluated *in vitro* and pulmonary metastasis was investigated *in vivo*. Mediators of cell signaling pathways were also examined following suppression of CD151 expression. Overall survival for patients with low versus high CD151 expression level was 94 vs. 41 months (*p*=0.0451). CD151 expression in osteosarcoma cells with high metastatic potential was significantly higher than in those with low metastatic potential (*p*<0.001). shRNA-mediated silencing of CD151 did not influence cell viability or proliferation; however, cell adhesion, migration and invasion were all inhibited (all *p*<0.001). In mice inoculated with shRNA-transduced osteosarcoma cells, the number and size of lung metastatic lesions were reduced compared to the mice inoculated with control-shRNA transduced cells (*p*<0.001). In addition, CD151 knockdown significantly reduced Akt, p38, and p65 phosphorylation as well as focal adhesion kinase, integrin β1, p70s6, and p-mTOR levels. Taken together, CD151 induced osteosarcoma metastasis likely by regulating cell function through adhesion signaling. Further studies are necessary to fully explore the diagnostic and prognostic value of determining CD151 expression in osteosarcoma patients.

## INTRODUCTION

Osteosarcoma is the most common primary bone tumor in adults and the third most common solid tumor in pediatrics with a 5-year survival rate of 60-70% [1]. However, a recent multicenter study analyzing osteosarcoma in Asian adults >40 y revealed that nearly 11% presented with initial metastasis, and response to neoadjuvant chemotherapy was classified as poor in 73% of those receiving it [2]. Patients with metastasis are less sensitive to chemotherapy with 5-year survival rate of 20-40% [1]. It is important to investigate mechanism underlying osteosarcoma metastasis and develop treatment strategy afterward.

The transmembrane protein, CD151, is a member of the transmembrane 4 superfamily, which consist of two extracellular domains, an intracellular N terminus, a loop and a C terminus [3, 4]. CD151 forms tetraspanin-enriched microdomains (TEMs) and may act on growth factor receptors and the laminin receptor, integrin, via its extracellular domain. It also interacts with intracellular signaling molecules, including phosphatidylinositol-4-OH kinase (PI4K), protein kinase C (PKC) and cytoskeleton proteins, through its intracellular domain, mediating cell-to-cell interactions or cell-to-extracellular matrix interactions. CD151 is closely related to the progression of breast, prostate and colon cancer, promoting metastasis [5-11]. In prostate cancer cells, CD151 is associated with increased invasiveness and lymphangiogenesis [12]. In addition to CD151 being a putative diagnostic and prognostic marker, its value as a therapeutic target has been shown in studies of CD151-specific antibodies [13]. Specifically, a monoclonal antibody, mAb 1A5, that targets CD151 inhibited fibrosarcoma and squamous cell skin cancer metastasis *in vivo* [14, 15]. Other antibodies against CD151, including 50-6, SFA1.2B4 and 1A5, significantly inhibit metastasis through suppression of angiogenesis, cell migration and invasion as well as intravascular permeability [4, 15-17].

We have previously shown that CD151 was highly expressed on the membrane of MG-63 osteosarcoma cells as compared to human hFOB1.19 osteoblastic cells [18]. To further investigate its role in osteosarcoma metastasis, CD151 expression was evaluated in paired osteosarcoma cells with high (LM8 and MG63.2 cells) and low (Dunn and MG63 cells) metastatic potentials. In addition, CD151 expression was silenced in osteosarcoma cells with high metastatic potential (ΔCD151 cells), and the adhesion, migration and invasion of these cells were subsequently evaluated. ΔCD151 cells were also inoculated into the primary osteosarcoma orthotopic model, and the pulmonary metastasis was assessed along with the mechanism underlying osteosarcoma metastasis. This study aimed to confirm the positive relationship between osteosarcoma metastasis and CD151 expression, and to examine the role of CD151 on osteosarcoma cell migration and invasion.

## RESULTS

### CD151 expression in human osteosarcoma is conversely associated with patient survival

**Figure 1:**
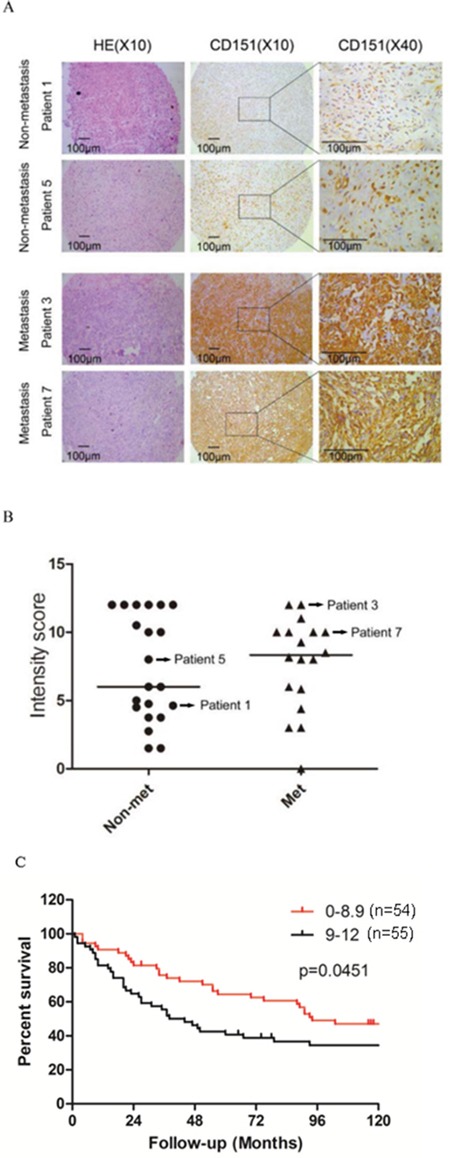
CD151 protein expression in human osteosarcoma **A.** Representative images from the immunohistochemical analysis of CD151 protein levels in tumor samples. **B.** Comparison of CD151 immunoreactivity in osteosarcoma tissues from non-metastatic patients and metastatic patients. Dash lines in the figure show the median values. **C.** Kaplan-Meier curves with log-rank tests were performed to investigate the differences of survival rate between patients with low (1-8.9; n=54) and high (9-12; n=55) CD151 levels (*p*=0.0451).

To evaluate the clinical significance of CD151 expression in human osteosarcoma, IHC analyses were conducted in two independent tumor tissue microarrays. TMA 1 consists 39 patients of which the demographic data and clinical characteristics of all patients are listed in Table 1. Of the osteosarcoma patients analyzed, 21 did not have metastases while 18 patients had metastasis. CD151 was only weakly expressed in the normal human muscle tissue (data not shown). In contrast, CD151 immunoreactivity was detected in a wide range of intensities in osteosarcoma tissue samples (Figure 1A). The median immunoreactive score (IRS) for CD151 expression in the 21 patients without metastasis was 6.0 (IQR in 4.5-12.0), and was 8.3 (IQR in 5.8-10.0) in the 18 patients with metastasis; however, the difference did not reach a statistical significance (*p*=0.757, Figure 1B).

**Table 1:** Clinical characteristics of the osteosarcoma patients

	n=39
Gender	
Male	23 (59.0%)
Female	16 (41.0%)
Tumor metastasis	
Non-metastasis	21 (53.8%)
Metastasis	18 (46.2%)^*^
Primary site	
Ilium	5 (12.8%)
Humerus	4 (10.3%)
Femur	18 (46.2%)
Tibia	8 (20.5%)
Others	4 (10.3%)
Score	
≤5	13 (33.3%)
5-10	10 (25.6%)
≥10	16 (41.0%)

* There were 14 lung metastases.

In TMA 2, containing a larger patient cohort and longer follow-up time, we found that the overall survival of patients with low CD151-expressing tumors (levels 1-8.9, n=54) was significantly higher than those with high-expressing tumors (levels 9-12, n=55) (*p*=0.0451) with median survival times of 94 months and 41 months, respectively (Figure 1C). In addition, the 10-year survival rate was 47.05% in the low-level group versus 34.49% in the high-level group.

### CD151 expression is associated with the metastatic potential of osteosarcoma cell lines

**Figure 2:**
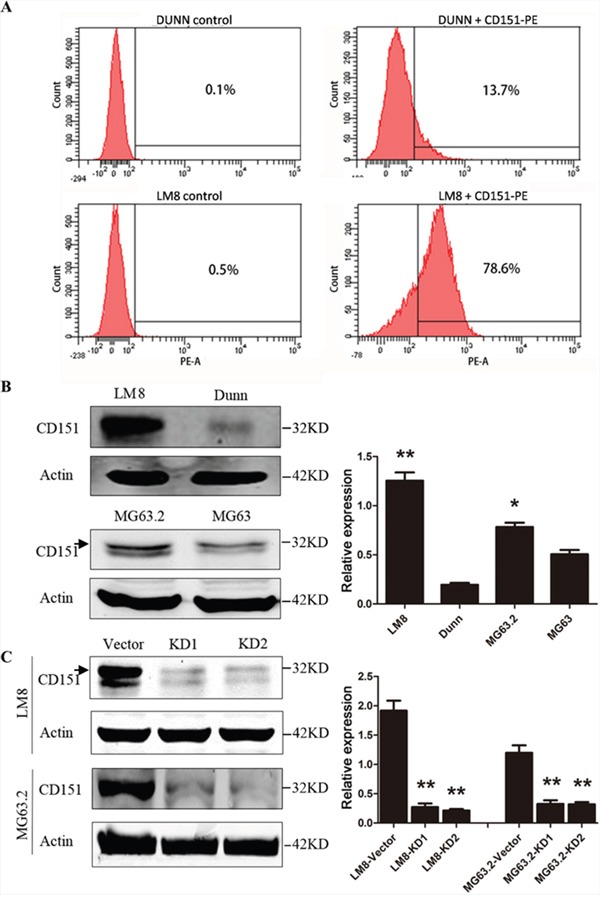
CD151 protein expression in osteosarcoma cell lines **A.** Detection of CD151 expression on the membranes of osteosarcoma cell lines, Dunn and LM8 cells, as determined by flow cytometry. **B.** Detection of CD151 expression in the osteosarcoma cell lines, LM8, Dunn, MG63, and MG63.2) by Western blot analysis. *LM8 and MG63.2 cells had significantly higher CD151 expression than Dunn and MG63 cells (*p*<0.001). **C.** LM8 and MG63.2 osteosarcoma cell were transduced with lentiviral vectors to express shRNA sequences targeting CD151 transcript (KD1 and KD2) or with empty lentiviral vectors (vector control). The expression of CD151 was determined by Western blotting. *Compared with vector control group, CD151 expression in LM8 and MG63.2 cells was significantly reduced (all *p*<0.001). Quantitative data are presented by mean and SD of three independent experiments. An arrow indicates the main band of CD151 in Western blot analysis.

The murine LM8 osteosarcoma cell line, a derivative the Dunn cell line, has higher metastatic potential than Dunn cells [19]. Flow cytometry analysis revealed that the proportion of cells with CD151 surface expression was 78.1±0.95% in LM8 cells, which was significantly higher than 13.6±0.4% in Dunn cells (P<0.001) (Figure 2A). Western blot analysis also confirmed that total CD151 expression was significantly higher in LM8 cells than that in Dunn cells (Figure 2B). Similarly, human MG63.2 cells, a derivative of MG63 cells with a higher metastatic potential [20], had significantly higher CD151 expression than MG63 cells (*p*<0.001) (Figure 2B). These findings suggested that CD151 expression is increased in cells with high metastatic potential.

Because LM8 and MG63.2 displayed significantly higher expression of CD151, these cell lines were selected as our representative models for subsequent functional analyses. Both osteosarcoma cell lines were transduced with sh-CD151-expressing lentiviral vectors (KD1 and KD2) or with the empty lentiviral vector (vector control). Compared with vector control group, CD151 expression in LM8 and MG63.2 cells was reduced by 87.5% (1.92 in vector vs. 0.27 in KD1 and 0.21 in KD2, *p*<0.001) and 72.5% (1.2 in vector vs. 0.33 in KD1 and 0.32 in KD2, *p*<0.001) (Figure 2C). Thus, ΔCD151 LM8 and MG63.2 cells were successfully established.

### CD151 did not alter osteosarcoma cell viability, cell cycle progression, and apoptosis

**Figure 3:**
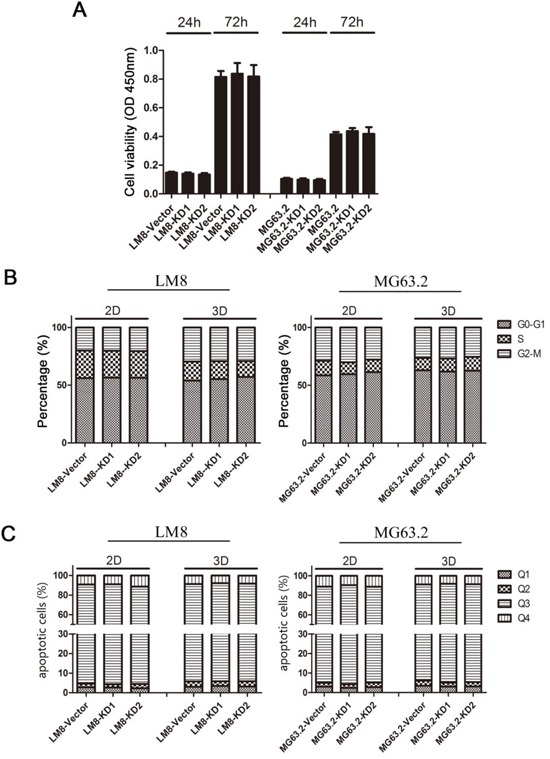
Effects of CD151 knockdown on LM8 and MG63.2 cell viability, cell cycle progression and apoptosis **A.** Cell viability was assessed in vector, KD1 or KD2 cells by MTT assays at 24 and 72 h after the cells were seeded in 96-well culture dishes using flow cytometry. **B, C.** Cells were seeded in normal (2D) 6-well culture dishes and three-dimensional (3D) 6-well culture dishes. After 24 h, cells were harvested for analysis of (B) cell cycle progression or (C) apoptosis. Each bar represents the mean value and the SD value (standard deviation) of five wells. Quadrant 1 (Q1) represents necrotic cells, quadrant 2 (Q2) late apoptotic cells, quadrant 3 (Q3) intact cells, and quadrant 4 (Q4) early apoptotic cells.

To investigate the biological function of CD151 in osteosarcoma, we next evaluated the viability, cell cycle progression, and apoptotic rates of the various ΔCD151 LM8 and MG63.2 cells. As shown in Figure 3A, CD151 silencing did not alter cell viability at 24 and 72 h. Analysis of the cell cycle distribution by flow cytometry revealed that the proportions of KD1 and KD2-expressing LM8 and MG63.2 cells in sub-G1, G0/G1, S and G2/M phases show no difference to that observed for the vector control cells (Figure 3B). Similar results were obtained for KD1, KD2 and vector-expressing LM8 and MG63.2 cells cultured in 3D (Figure 3B). The proportion of apoptotic cells in each cell type is shown in Figure 3C. In both adherent cells and suspended cells, the proportion of apoptotic cells remained unchanged after CD151 knockdown. Thus, suppression of CD151 expression did not affect osteosarcoma cell viability, cell cycle progression or apoptosis.

### CD151 silencing reduces osteosarcoma cell-cell attachment and adhesion

**Figure 4:**
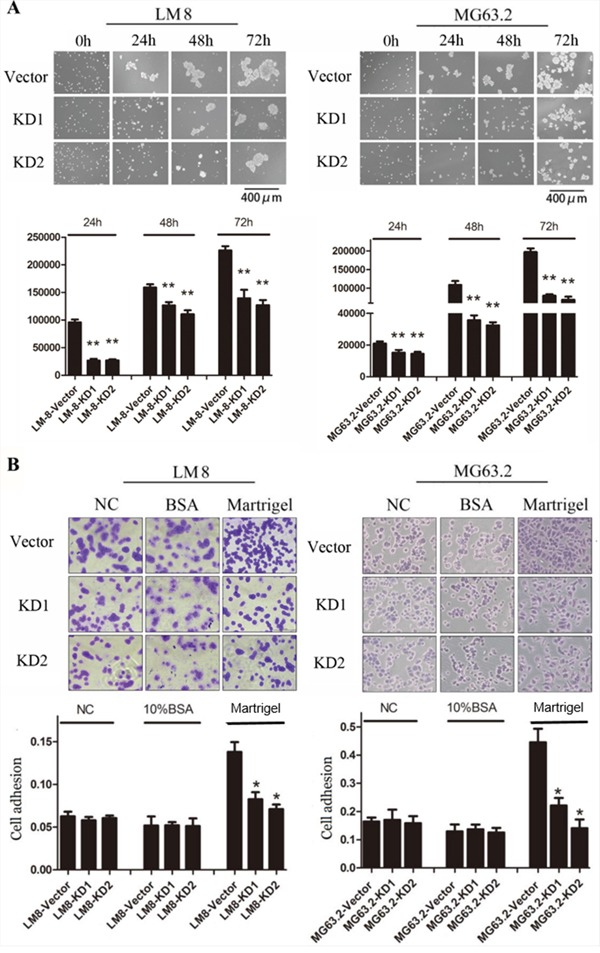
Effects of CD151 knockdown on osteosarcoma cell-cell attachment and cell adhesion **A.** Representative images of cell-cell attachment were shown for cells expressing the control or CD151 shRNA after 0, 24, 48 and 72 h of Poly-HEMA-coated non-adherent three-dimensional culture. Average colony areas in multiple random fields were quantified. **B.** Equal numbers of the indicated cells were seeded in 96-well plates precoated with Matrigel, BSA or nothing for 45 min. The attached cells were fixed with 95% ethanol, stained with crystal violet, and visualized using a phase-contrast microscope. Scale bar: 100 μm. Absorbance was measured at 595 nm after the crystal violet was dissolved with 33% glacial acetic. Quantitative data are presented by mean and SD of three independent experiments. ^*^p<0.01, indicates a statistically significant difference compared to the vector control.

CD151 is a cell membrane molecular related to adhesion, which is important for cancer cell motility. In order to test its role in osteosarcoma, we next studied whether CD151 silencing reduced the cell-cell attachment or cell adhesion of osteosarcoma cells. Analysis of the cell-cell attachment spheroid area as determined by Image J showed that cell-cell attachment was significantly reduced in the KD1 and KD2-expressing LM8 and MG63.2 cells at 24, 48, and 72h as compared to the vector control cells (*p*≤0.001; Figure 4A). As shown in Figure 4B, LM8 and MG63.2 osteosarcoma cell adhesion following CD151 knockdown was significantly reduced compared with vector control cells (*p*<0.001).

### Suppression of CD151 expression reduced osteosarcoma cell migration and invasion

**Figure 5:**
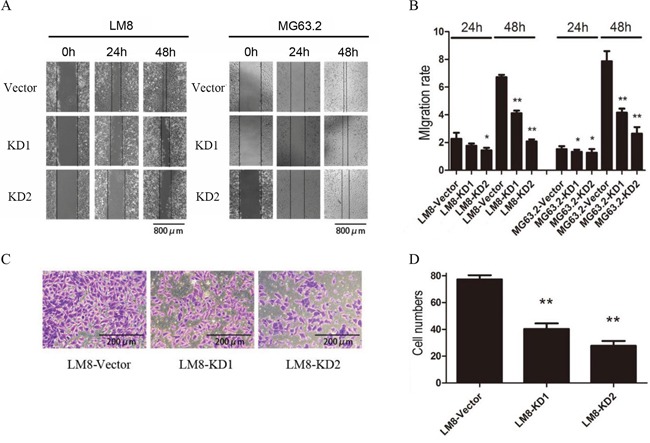
CD151 knockdown inhibits osteosarcoma cell migration and invasion **A.** Representative images of LM8 and MG63.2 cell migration. **B.** The migration rate was determined by measuring the mean change in the gap width at three representative sites. Quantitative data are presented by mean and SD of three independent experiments. **C.** Representative images of Matrigel invasion by LM8 cells. **D.** Comparison of invaded cell numbers among groups. ^*^indicates a statistically significant difference compared to the vector control (all *p*<0.001). ^**^indicates a statistically significant difference compared to the KD1 group (*p*<0.001 for LM8 cells and *p*=0.013 for MG63.2 cells).

To further validation the relation between CD151 and cell motility, the effect of CD151 suppression on osteosarcoma cell migration and invasion was next examined. As shown in Figure 5A and 5B, the migration rate was significantly lower in both KD1 and KD2-expressing LM8 and MG63.2 cells at 48 h compared to the vector control (all *p*<0.001). Similarly, the number of invaded CD151-KD1 and CD151-KD2 cells was significantly reduced compared with the vector control group (*p*<0.001; Figure 5C, 5D).

### CD151 silencing alters osteosarcoma cell signaling

**Figure 6:**
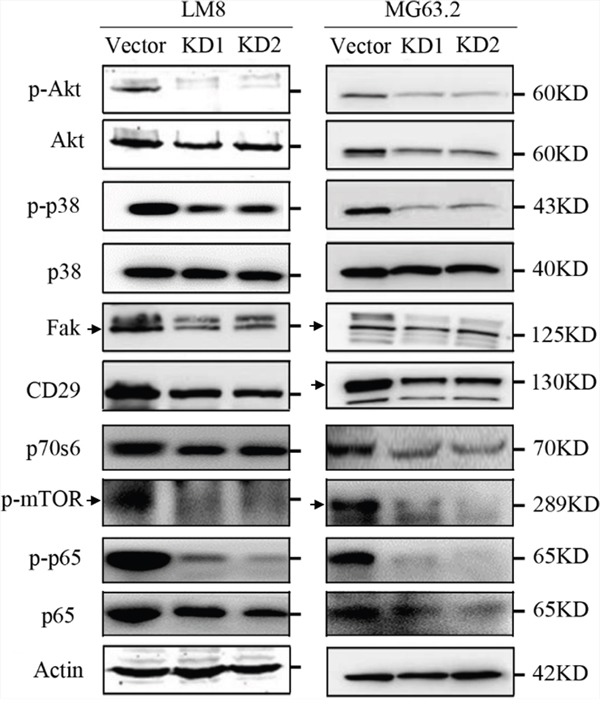
CD151 silencing alters osteosarcoma cell signaling Phosphorylated versus total protein ratios of Akt, p38, and p65 as well as FAK, CD29, p70s6, and p-mTOR levels in LM8 and MG63.2 were determined by Western blot analysis. An arrow indicates the main band of each molecule in Western blot analysis.

Because CD151 interaction with extracellular matrix or intercellular junctions can alter the migration of malignant cells through cell signaling [13], the effects of its suppression on cell signaling molecules with known roles in cell motility and migration, including the Akt and p38 signaling pathways [21, 22], were detected by western blot. Protein levels of Akt, phosphorylated Akt (p-Akt), p38, phosphorylated p38 (p-p38), focal adhesion kinase (FAK), CD29 (integrin β1), p70s6, p-mTOR, p-p65, and p65 were analyzed. In LM8 cells, CD151 knockdown reduced Akt phosphorylation; both Akt and p-Akt levels were decreased in MG63.2 cells following CD151 knockdown. In addition, p38 phosphorylation in LM8 and MG63.2 cells were both reduced with CD151 knockdown. CD151 knockdown also reduced FAK, CD29, p70s6, p-mTOR, p-p65 and p65 expression in both LM8 and MG63.2 cells (Figure 6).

### Depletion of CD151 attenuates pulmonary metastasis *in vivo*

**Figure 7:**
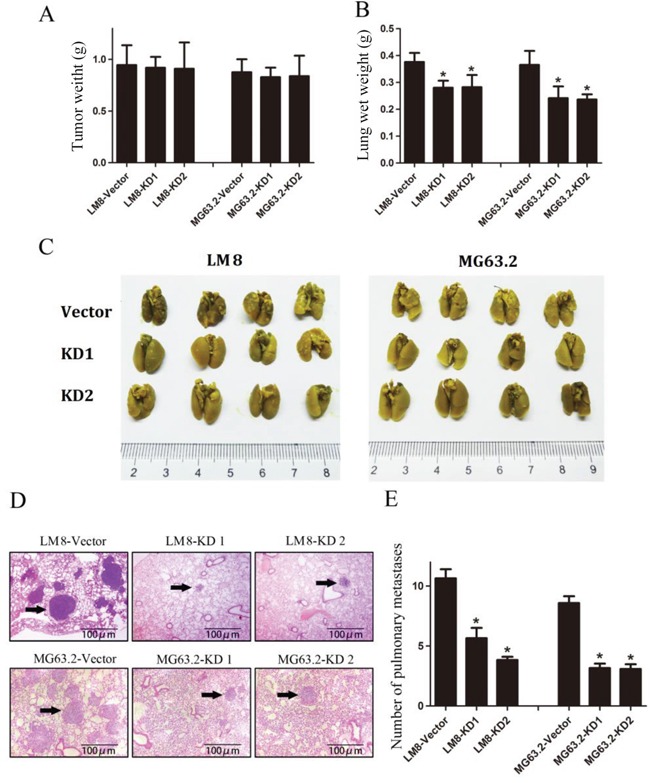
CD151 knockdown suppresses osteosarcoma pulmonary metastasis *in vivo* **A.** Tumor weights at 30 days after tibial injection of LM8 (left) and MG63.2 (right) cells. **B.** Weight of the lungs at 30 days after tibial injection of LM8 cells (left) and MG63.2 cells (right). **C.** Representative photographs of pulmonary metastatic foci produced 30 days after tibial injection of LM8 cells (left) or MG63.2 cells (right). **D.** Representative HE analysis of lung metastases from mice injected with LM8 cells groups (top) or MG63.2 cells groups (bottom). Arrows indicate metastatic foci. **E.** Numbers of pulmonary metastatic foci. Quantitative data are represented as the mean and SD (n=5). ^*^indicates a statistically significant difference compared to the vector control (all *p*<0.001).

We next investigated whether CD151 ablation attenuated pulmonary metastasis *in vivo* using an orthotopic osteosarcoma tibial model with pulmonary metastasis model. As shown in Figure 7A, there was no significant difference in the primary tumor weights among the groups in mice either inoculated with LM8 cells or MG63.2 cells. However, the lung weights of mice inoculated with KD1 and KD2-expressing LM8 and MG63.2 cells were significantly reduced as compared to the vector control group (all *p*<0.001; Figure 7B).

Representative photographs of the pulmonary metastatic foci were shown in Figure 7C, and representative lung HE images of the metastases were shown in Figure 7D. Analysis of the number of metastatic foci in the lungs revealed that there were fewer in mice inoculated with CD151-KD1 and CD151-KD2 expressing LM8 and MG63.2 cells compared with those inoculated with control cells (all *p*<0.001; Figure 7E), suggesting that CD151 expression contributes to the progression of pulmonary metastasis.

## DISCUSSION

CD151 expression is closely related to the progression of breast, prostate and colon cancer [5-10]; its expression is positively associated with the metastasis of breast [8, 23-25] and prostate [5, 11, 26] cancers. However, its role in osteosarcoma was relatively unknown. This study presents clinical and experimental evidence of the role of CD151 in osteosarcoma metastasis. In the present study, IHC analysis revealed that patients with metastasis had greater CD151 expression as compared to those without metastasis although the difference was not significant. Consistent with this observation, CD151 knockdown markedly reduces osteosarcoma cell migration and invasion, but not cell viability, cell cycle progression or apoptosis. Furthermore, cell-cell attachment and adhesion in osteosarcoma cells were markedly decreased with CD151 knockdown as was the phosphorylation of p38 and Akt and the expression levels of FAK and integrinβ1. Finally, an *in vivo* pulmonary metastasis model revealed that inoculation of shCD151-expressing osteosarcoma cells, which are originally highly metastatic, produced few lung metastases as compared to the vector control cells. Taken together, our results demonstrate that CD151 knockdown attenuates osteosarcoma tumor pulmonary metastasis and points to a new role for CD151 as a regulator of cell adhesion between tumor cells and the extracellular matrix.

Previous datasets by Kobayashi, Guenther, and Buddingh (http://hgserver1.amc.nl/cgi-bin/r2/main.cgi?&species=hs) have suggested a positive correlation between CD151 expression and tumor metastasis (Supplementary Figure 1). In addition to its role in cancer progression and metastasis, studies have shown that CD151 promotes tumor neovascularization [13] and tumor growth [4]. Furthermore, elevated CD151 expression was previously linked to poor prognosis in human lung [27] and prostate cancers [5]. Furthermore, our previous work has suggested that CD151 may represent a new biomarker in the detection and diagnosis of human osteosarcoma [18]. In the present study, slightly higher but not significant expression level of CD151 was detected in patients with metastasis than patients without, which may be due to the small sample size. Larger patient cohort clearly suggested that CD151 expression correlates with poorer prognosis. We also analyzed the effect of CD151 over-expression *in vivo*, but observed no differences (data not shown), suggesting that baseline levels of CD151 may be sufficient to induce metastasis. Furthermore, CD151 silencing suppressed osteosarcoma metastasis without altering osteosarcoma cell viability or proliferation *in vitro* as well as LM8 or MG63.2 tumor morphology and growth *in vivo*. These results are consistent with a recent report by Zhang et al. [28] in which CD151 knockdown inhibits metastasis in one osteosarcoma cell line with continuous KRAS activation (143B). Similarly, in CD151 knockout mice, the number of metastatic lesions in the lung was about 25% of that observed in the control group although the area of metastatic lesions remained unchanged [29-31]. Hence, CD151 most likely affects the later stages of osteosarcoma progression, in which cells invade into surrounding tissue and encounter the extracellular matrix. In addition, CD151 is regarded as a potential therapeutic target based on its role in promoting tumor progression, and several groups have proposed and developed antibody-based immunotherapies against CD151 [15] while others have reported soluble large-loop proteins or RNAi technology to inhibit CD151 [4, 32]. However, the clinical benefits of these therapeutic strategies have yet to be determined.

Consistent with the putative role of CD151 in human osteosarcoma metastasis, we observed that CD151 expression in osteosarcoma cells with high metastatic potential was significantly higher than that in cells with low metastatic potential. Furthermore, previous studies have shown that CD151 overexpression increased the migration and invasion of malignant cells, which was ameliorated with CD151 knockdown [24, 33, 34]. These findings were consistent with the present study in which CD151 silencing inhibited LM8 cell migration and Matrigel invasion as well as MG63.2 cell migration. Given that miR-152 was reported to target CD151 expression in gastric cancer, repressing gastric cancer cell proliferation and motility [35], further studies will evaluate the role of this particular microRNA in regulating CD151 expression in osteosarcoma.

The current understanding of tumor invasion is that there are two modes of tumor cell movement: one is the proteolysis-guided mesenchymal movement induced by Rho/ROCK, and the other is the actomyosin-driven amoeboid movement by proteases, particularly matrix metalloproteinases (MMPs) [36]. CD151-deficient cells exhibited decreased levels of the Rho family with integrins and small GTPase activation [6, 37], resulting in the decreased invasion and migration of tumor cells [36, 38]. Cell migration is also biphasic with respect to adhesion strength [38]. CD151 is known to support adhesion strengthening [29], which is consistent with the present study in which ablation of CD151 inhibited the stable cell-cell attachments in LM8 and MG63.2 osteosarcoma cells. These findings suggest that CD151 silencing reduced the intercellular adhesion in a similar manner as that reported for breast cancer intercellular adhesion [8, 14, 23, 39, 40]. In addition, the adhesion of the osteosarcoma cells to the extracellular matrix was compromised upon CD151 silencing. Because elevated adhesion to neighboring cells and extracellular matrix may increase cellular migration and subsequently influence the invasion of malignant cells, the reduced metastasis observed with ablation of CD151 may be mediated through suppression of osteosarcoma cell adhesion.

CD151 interaction with extracellular matrix or intercellular junctions can alter the migration of malignant cells via altering cell signaling events [13] that results in the upregulation of enzymes involved in the degradation of extracellular matrix [29, 41]. Both PI3K-Akt and p38-MAPK induce cellular migration and invasion, as well as capillary formation and intravascular permeability [21, 22]. In the present study, p38 and Akt phosphorylation was reduced significantly after CD151 silencing. However, further studies are required to determine if these signaling pathways mediated the reduction in metastatic potential observed with CD151 silencing.

CD151 may directly interact with α3β1, α6β4, α6β1 and β1 integrins to alter the adhesion with adjacent cells and extracellular matrix [39, 42-45]. In B16BL6 murine melanoma cells, interaction of CD151 with α3β1 integrin was reduced with its glycosylation, subsequently altering cell migration and invasion [46]. Tyrosine phosphorylation of the non-receptor tyrosine kinase, FAK, in response to cell adhesion represents an early event in integrin signaling [47]. FAK, which alters the PI3K/Akt, MAPK/p38, and phospholipase C pathways [41, 47-51], is essential for cell migration and plays an important role in tumor metastasis [48, 52]. In the present study, the protein levels of integrin β1 and FAK were reduced significantly following CD151 silencing. The reduced integrin β1 and FAK protein levels with CD151 silencing may have resulted in the altered phosphorylation of Akt and p38 and subsequent reduction in cell adhesion and metastasis; however, further studies are required to determine the effects were mediated by additional pathways. In addition, the spheroid size was reduced following CD151 knockdown after 24 h which was accompanied with decreased cell attachment. This is consistent with a previous study, which reported that CD151 alters α6β1 integrin adhesion strengthening [50].

Taken together, CD151 promotes the migration, invasion, and metastasis of osteosarcoma by altering adhesion, suggesting that it may prove to be a critical diagnostic and/or prognostic marker as well as therapeutic target. However, the specific molecular mechanism underlying the regulatory role of CD151 in the metastasis of osteosarcoma and the efficacy of therapy targeting CD151 remain to be elucidated in further studies.

## MATERIALS AND METHODS

### CD151 expression by immunohistochemical (IHC) staining on tissue microarrays

TMA-1 contains samples from patients treated in Shanghai General Hospital. TMA-2 contains samples from patients treated in Massachusetts General Hospital, Boston. All patient samples and clinical data in this study were retrieved after patient consent and Institutional Review Board (IRB) approval from each institution were obtained. IHC analyses of a tumor tissue array (TMA) and human osteosarcoma tumor tissues were conducted essentially as described previously [16]. In brief, antigens were retrieved using a decloaking chamber in the presence of 0.01M EDTA buffer (pH 9.0). After blocking in goat serum, TMA slides were incubated with a CD151-specific monoclonal antibody (Life Span BioSciences, Seattle, WA, USA) followed by incubation with a biotinylated secondary antibody. The reaction was developed using a DAB Kit (BD Bioscience, Franklin Lakes, NJ, USA), and the tissues were counterstained with hematoxylin. The immunoreactive score (IRS) was evaluated on the basis of staining intensity and the percentages of positively stained areas by three independent staff members as previously described [53]. The proportion of immunopositive tumor cells was evaluated. The intensity of immunostaining was evaluated by using four scores as follows: strong immunopositivity of the tumor cells in low-power view (×40), 3+; weak immunopositivity in low-power view but obvious staining in high-power view (×400), 1+; median intensity between 1+ and 3+, 2+; and absence of immunostaining, 0. The percentage of immunopositive tumor cells is also evaluated and scored as 0(<5%), 1(5-25%), 2(25-50%), 3(50-75%), 4(75-95%). Final scored was multiplied by immune-intensity score and immune-percentage score.

### Cell lines and culture

The murine LM8 osteosarcoma cell line with high metastatic potential and its parental cell line Dunn with low metastatic potential were originally established by Prof. Tatsuya Asai (Department of Orthopaedic Surgery, Osaka Medical Center for Cancer and Cardiovascular Diseases, Osaka, Japan). The human MG63.2 osteosarcoma cell line with high metastatic potential was previously established and characterized [20, 54], and the MG63 human osteosarcoma line with low metastatic potential was purchased from ATCC (Manassas, VA, USA). DNA fingerprinting was performed to rule out cell culture contamination. All cells were cultured in high glucose Dulbecco's Modified Eagle's Medium (DMEM-h) supplemented with 10% fetal bovine serum, 100 U/mL penicillin, and 100 μg/mL streptomycin (all from Invitrogen, Carlsbad, CA, USA) in a humidified incubator at 37°C in 5% CO_2_. Cells in logarithmic growth phase were harvested and used in the following experiments.

### Preparation of LM8 and MG63.2 osteosarcoma cells with stable expression of CD151 short-hairpin RNA (shRNA)

The specific shRNA sequences targeting CD151 were determined according to the WI siRNA Selection Program (http://sirna.wi.mit.edu/). For each cell line, the following two candidate sequences for siRNA knockdown of CD151 were selected: LM8 cells, gaacctgttacgcttgtactt (KD1) and cagctgctgtaagactatggt (KD2); MG63.2 cells, gcctcaagtacctgctgttta (KD1) and gagaccatgcctccaacatct (KD2). DNA sequences that expressed the above siRNA sequences and contained the appropriate restriction enzyme cleavage sites were synthesized for the construction of a lentivirus expressing CD151 shRNA. Subsequently, this lentivirus was transfected into 293T cells in the presence of plasmids, followed by lentivirus packaging. The supernatant was harvested after 48 h and filtered through a 0.45-μm filter. LM8 and MG63.2 osteosarcoma cell were transduced with lentiviral vectors expressing each shRNA sequence targeting the CD151 transcript (KD1 and KD2) or with empty lentiviral vectors (vector control). Cells with stable expression of CD151 shRNA or empty lentiviral vector were screened with 2 μg/mL puromycin.

### Western blot assay

Cells were harvested, washed twice with PBS, and lysed with RIPA Lysis Buffer (Beyotime Institute of Biotechnology, Shanghai, China) with protease inhibitors (Thermo Fisher Scientific). Tumor tissues retrieved from −80°C storage were rapidly immersed in liquid nitrogen followed by lysis with RIPA Lysis Buffer and protease inhibitors. Protein concentrations were determined with a Pierce BCA Protein Assay Kit (Thermo Fisher Scientific). Total protein (50 μg/lane) was separated by 10% SDS-PAGE and was transferred to nitrocellulose membranes. After blocking for 60 min with 5% milk solutions prepared in PBS, the membranes were incubated overnight at 4°C with 1:1000 dilutions of the following primary antibodies: CD151 (#5901), FAK (#2146-1), and CD29 (#1798-1; all from abcam, Cambridge, UK), Akt (#4685), p-Akt (#4060), p38 (#8690), p-p38 (#4511), mTOR (#5536), p70S6 (#9208), p65 (#8242), and p-p65 (#3033; all from Cell Signaling Technology, Danvers, MA, USA). After the membranes were washed, they were incubated for 1 h with the appropriate peroxidase-conjugated secondary antibody (1:1000 dilution). Membranes were developed using the Odyssey Two-Color Infrared Laser Imaging System (LI-COR Biosciences, Lincoln, NB, USA). The signal generated by actin (#4973; Cell Signaling Technology) was used as an internal control.

### Detection of cell cycle and apoptosis by flow cytometry

Osteosarcoma cells were seeded into 6-well plates (3×10^5^ cells/well) and incubated in a humidified environment of 5% CO_2_ at 37°C for 24 h. To determine cell cycle progression, cells were harvested, washed in PBS twice, incubated in 300 μL of 1× binding buffer with propidium iodide (PI, Sigma, St. Louis, MO, USA), NP-40, and RNase A (BD Biosciences, San Jose, CA, USA) at room temperature for 15 min. To evaluate apoptosis, the cells were incubated in 100 μL of 1× binding buffer with annexin-V/PI double staining solution (BD Biosciences) at room temperature for 15 min. The stained cells were analyzed by flow cytometry. The proportion of cells in the different stages of the cell cycle or those that were apoptotic or necrotic were calculated using Mod-Fit LT software (Verity Software House).

### Detection of cell viability

Cells (3×10^3^ cells/well) were seeded into 96-well plates and cultured overnight. After 24 or 72 h, 20 μL of MTT (5 mg/mL, Sigma) was added into each well for another 4 h. The supernatant was removed, and 150 μL of DMSO was added into each well. The absorbance was then measured using a model ELX800 Micro Plate Reader (Bio-Tec Instruments, Winooski, VT, USA) at 490 nm.

### Detection of CD151 surface expression by flow cytometry analysis

Osteosarcoma cells were seeded into plates (8×10^5^ cells/well) and incubated in a humidified environment of 5%CO_2_ at 37°C. After 24 h, cells were harvested and washed twice in PBS. The cell suspensions (1×10^6^ cells/40μl) were incubated with 10 μL of CD151-PE (R&D, Minneapolis, MN, USA) in the dark at 2-8°C for 45 min. Following washing in PBS twice, 500 μL of PBS was added to prepare single cell suspensions, and flow cytometry analysis was performed using a BD-FACS Calibur (BD Biosciences).

### Analysis of cell-cell attachment

Poly-2-hydroxyethyl acrylate (Sigma) was added to 6-well plates (1 mL/well), which were air-dried. The osteosarcoma cells were seeded into plates (3×10^5^ cells/well) and incubated in a humidified environment of 5% CO_2_ at 37°C. Adherent cells were characterized by spherical growth in three-dimensional (3D) 6-well culture plates. Cell-cell attachments were photographed at 0, 24, 48 and 72 h using a phase-contrast microscope. Cell-cell attachments, which were presented as the average spheroid size (1 × 10^3^ m^2^) = the area/number of spheroid, in multiple random fields were quantified using Nikon NIS-Elements Advanced Analysis Software.

### Detection of cell adhesion

Matrigel (20 μL/well, BD Biosciences) and 10 mg/mL BSA (20 μL/well, Sangon Biotech, Shanghai, China) were added to 96-well plates, followed by incubation at 37°C for 0.5-1 h. Cells were seeded into 96-well plates (1×10^4^/well) and incubated at 37°C for 45 min. The suspended cells were removed, and the adherent cells were washed in PBS twice and fixed in 95% ethanol at room temperature for 10 min. Cells were air-dried and stained with crystal violet for 20 min. After the cells were washed and air-dried, 33% glacial acetic acid (Sangon Biotech, Shanghai, China) was used to dissolve the crystal violet, and optical density (OD) was measured at 595 nm. The cell adhesion rate was calculated as follows: cell adhesion rate = (OD_Experiment group_– OD_BSA group_)/OD_BSA group_.

### Detection of cell migration

Cells were seeded into 6-well plates (2×10^5^ cells/well). After 24 h, a scratch was made in the monolayer using a pipette, and the cells were washed twice in PBS. The cells were maintained in essential medium containing 2% FBS and were photographed at 0, 24 and 48 h to observe the cell migration. The cell migration rate was calculated as follows: cell migration rate = wound area at 0 h/wound area at a specific time point.

### Transwell invasion assay

Matrigel was added (50 μL/well) to the upper chambers of a Transwell followed by incubation at 37°C. After 30 min, the cells were seeded at 5×10^4^ cells/well. Medium containing 2% FBS was added to the upper chambers, and medium containing 10% FBS was added to the lower chambers, and the plates were incubated at 37°C for 24 h. Cells in the upper chambers were removed, and lower chambers were washed in PBS twice, fixed in 95% ethanol, air-dried and stained with crystal violet. Cells were photographed, and the number of cells in the lower chambers was determined.

### Animal experiments

C3H mice (inoculated with LM8 cells) and nude mice (inoculated with MG63.2 cells) aged 4 weeks were purchased from the Shanghai SLAC Experimental Animal Center (Shanghai, China). Mice were housed in a standard animal laboratory with free access to water and food. They were kept under constant environmental conditions with a 12-h light–dark cycle. All operations were performed under aseptic conditions. All the animal-related procedures were approved by the Institutional Animal Care and Use Committee (IACUC) of Shanghai, General People's Hospital of Shanghai.

### Mice tibial tumor models and pulmonary metastasis models

After the LM8 and MG63.2 cells were washed with cold PBS, they were suspended in cold PBS at a final concentration of 1 × 10^8^ cells/mL. LM8 cells (10 μL) were injected into the tibia of C3H mice, and MG63.2 cells (10 μL) were injected into the tibia of nude mice. Specifically, the cell suspensions were injected into the medullary cavities of the tibia. Mice were divided into the following three groups (10 mice per group): vector control group, KD1 group and KD2 group. All of the mice were euthanized at once the first mouse died. The lungs and legs were collected, fixed, embedded and sectioned for hematoxylin and eosin (HE) staining or IHC analysis. Histological examination and counting of the metastatic lesions were performed. Lungs containing metastases were immersed in Bouin solution to distinguish white tumor colonies from yellowish lung parenchyma. Metastasis was measured by visually counting the number of lesions in the entire mouse lung section for each mouse using Nikon NIS-Elements software (Nikon Corporation Instruments, Tokyo, Japan) at 10X magnification.

### Statistical analysis

The IRS for the 39 human osteosarcoma tissues had non-normal distribution and, thus, was expressed as median and inter-quartile range (IQR). The non-parametric Mann-Whitney test was performed to compare differences in IRS between patients with and without metastasis. Other continuous data were expressed as mean ± standard deviation (SD). One-way analysis of variance (ANOVA) was performed to compare the differences between groups; post-hoc tests with Bonferroni correction were performed to compare the pair-wise groups. For survival rates, Kaplan-Meier curves with log-rank tests were performed to investigate the differences of survival rate between patients with low (1-8.9) and high (9-12) CD151 levels. *P* < 0.05 was considered statistically significant. All experiments were repeated three times. The statistical analysis was performed using GraphPad Prism 5.0 (La Jolla, CA, USA).

## SUPPLEMENTARY FIGURE


