# Supplementary material for: CD151-mediated adhesion is crucial to osteosarcoma pulmonary metastasis
Source: Oncotarget. 2016 Aug 19;7(37):60623–38. doi: 10.18632/oncotarget.11380 (PMC5312406; doi:10.18632/oncotarget.11380)
Supplement: Supplementary file 1 [file oncotarget-07-60623-s001.pdf]

CD151-mediated adhesion is crucial to osteosarcoma pulmonary metastasis

SUPPLEMENTARY FIGURE

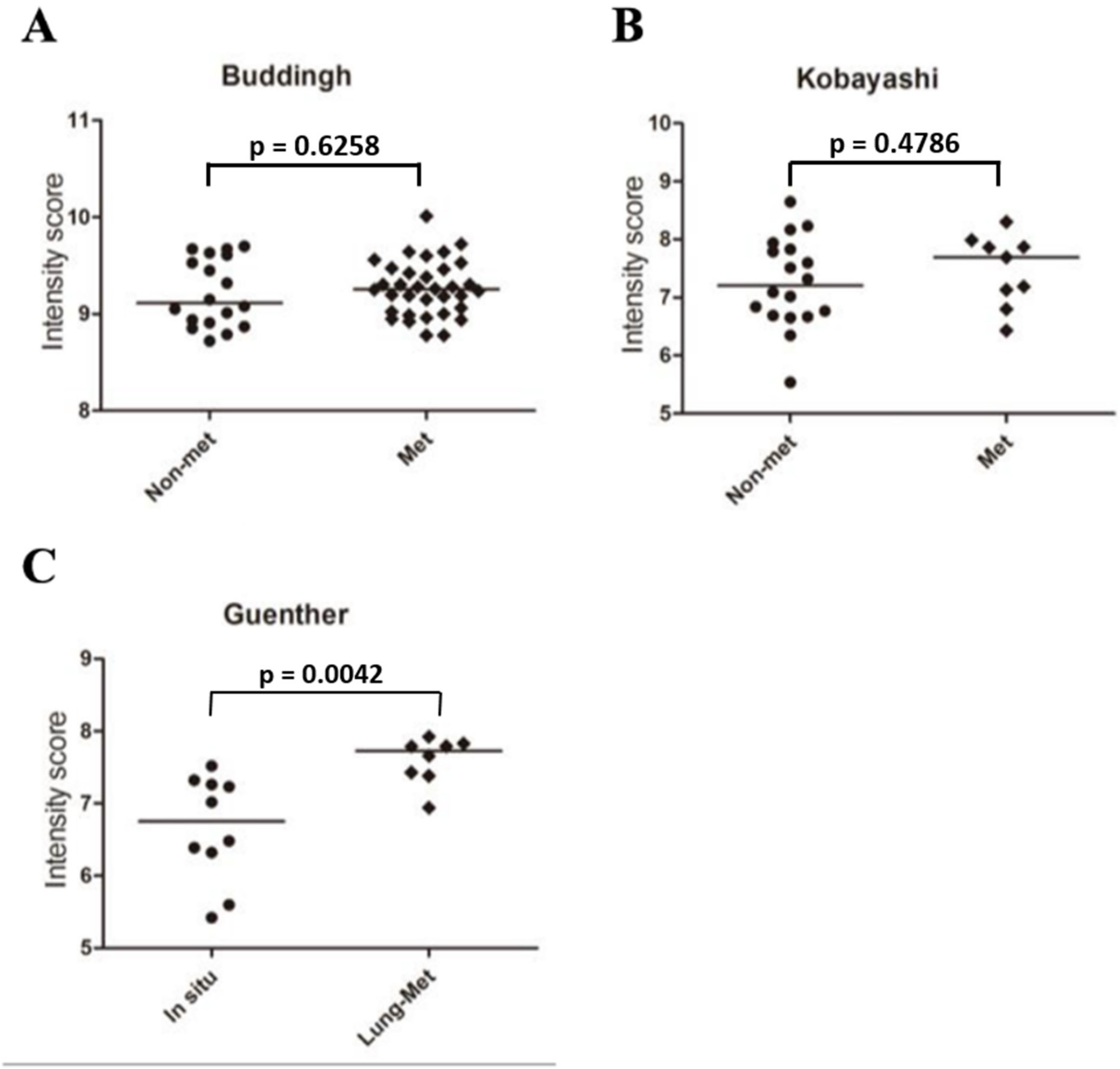

Supplementary Figure 1: Comparison of CD151 mRNA expression between non-metastatic and metastatic patients based on the data collected online. **A.** The Kobayshi, **B.** The Guenther database, and **C.** The Buddingh database.
